# Vision-Based Building Seismic Displacement Measurement by Stratification of Projective Rectification Using Lines

**DOI:** 10.3390/s20205775

**Published:** 2020-10-12

**Authors:** Jia Guo, Yang Xiang, Kohei Fujita, Izuru Takewaki

**Affiliations:** 1 International Research Institute of Disaster Science (IRIDeS), Tohoku University, Sendai 980-8572, Japan; 2 Department of Architecture and Architectural Engineering, Kyoto University, Kyoto 615-8540, Japan; xiang.y.aa@m.titech.ac.jp (Y.X.); fm.fujita@archi.kyoto-u.ac.jp (K.F.); takewaki@archi.kyoto-u.ac.jp (I.T.)

**Keywords:** seismic displacement measurement, vision-based system, projective rectification, vanishing line estimation

## Abstract

We propose a new flexible technique for accurate vision-based seismic displacement measurement of building structures via a single non-stationary camera with any perspective view. No a priori information about the camera’s parameters or only partial knowledge of the internal camera parameters is required, and geometric constraints in the world coordinate system are employed for projective rectification in this research. Whereas most projective rectifications are conducted by specifying the positions of four or more fixed reference points, our method adopts a stratified approach to partially determine the projective transformation from line-based geometric relationships on the world plane. Since line features are natural and plentiful in a man-made architectural building environment, robust estimation techniques for automatic projective/affine distortion removal can be applied in a more practical way. Both simulations and real-recorded data were used to verify the effectiveness and robustness of the proposed method. We hope that the proposed method could advance the consumer-grade camera system for vision-based structural measurement one more step, from laboratory environments to real-world structural health monitoring systems.

## 1. Introduction

Observing the vibration characteristics of building structures in a metropolitan area subjected to extreme loading (e.g., earthquake) with state-of-the-art sensing technologies has become a valuable method for structural safety assessments and design validations. Generally, the behavior of building structures could be evaluated in terms of two types: the acceleration response corresponding to the short period components of the external load and the displacement response corresponding to the long period components. The monitoring of the short-term as well as of long-term responses of building structures under severe loading conditions and verifying their spectral characteristics are both of importance in many cases. For instance, high-rise buildings far from the epicenter of the Great East Japan earthquake were reported to have been shaken intensively by the ground motion of short to long period components [1,2]. In practice, acceleration measurements are most convenient for use in structural dynamics applications. Nevertheless, monitoring the displacement responses of structures via the double integration of accelerations is always problematic due to the nature of signal processing: the integration constants cannot be determined, and thus only part of the response can be fully recovered. Besides, the numerical integration itself can also be inaccurate, based on the theory of error propagation [3]. With the recent development of sensing and robotic technologies, new generation displacement sensing techniques, including Global Positioning System (GPS), Speckle Photography, and laser Doppler vibrometer, provide significant advantages to supplement accelerometers for structural response monitoring. However, utilization of these techniques has to overcome many practical limitations such as the issue of obstructions, the requirement of high set costs, the need of stable platform, complex data interpretation, and hardly fully assessed accuracy [4,5,6].

Recently, with the aid of the digital image processing techniques and modern computer and information science, the field of vision-based displacement measurement techniques has attracted intensive attention in structural health monitoring, for its advantages of ease of operation and non-contact flexibility. A vision-based measurement system typically consists of one video camera or multiple cameras, and a computer. While most previous vision-based applications in structural response monitoring end up with producing satisfied displacement tracking results using stationary camera [7,8,9], which is not easy to ensure for field measurements during earthquakes or typhoons, limited research examined the effects of the camera motion on the measurement accuracy.

A first general approach to consider for freely moving camera is the computation of an optimal visual reconstruction of camera motion and 3D scene structure by extracting background point-based features within the un-deformed/rigid region of the view. A typical application of this method in structural vibration-based monitoring can be found in [10]. Challenging tasks during the reconstruction process involve the issue of matching ambiguities for repeated structures in the scene (e.g., regular building facade), and achieving sub-pixel measurement accuracy, since small deviations in the camera motion estimation might result in large measurement errors.

An alternative simplified approach to address this challenge was to explore partial or complete 3D metric information directly from the analysis of 2D geometric properties in the world system. When a projection of 2D world coordinates to 2D image coordinates is obtained, the effect of the camera motion can be directly removed by the projective rectification. This approach is always precise enough, easy to conduct and thus widely applied in laboratory experiments and field tests [11,12,13]. In order to improve the accuracy of image rectification, enough high-contrast natural regions of interest (ROIs) from parallel background world planes are required to compute the projective transformation for each image. However, recent research shows that these ROIs are always deficient in real-world applications [14,15,16].

Meanwhile, for a wide variety of man-made architectural building environments, *line segments* have been proven useful to achieve rich and reliable geometric information of the 3D structure in images, due to their abundance on man-made objects. Examples and applications of line-based vision task include facade detection [17], camera calibration [18], camera pose estimation [19], stereo SLAM [20], etc. State-of-the-art technologies with high-precision as well as full automatization in line detection [21], line matching [22], vanishing point detection [23], etc., provide solid basic steps for the completion of these tasks.

In this paper, we propose a novel vision-based seismic displacement measurement method designed for building structures. We address the problem of projective transformation estimation by extracting line-based, rather than point-based, geometric information from the architectural building environments. Since line segments are always abundant in building structures, robustness estimation techniques for automatic projective/affine distortion removal can be applied in a more practical way. The main advantages of our proposed algorithm include:

- This study addresses the problem of extracting structural dynamic displacement information from a *single*, *uncalibrated* camera;
- There is no need for stationary cameras and cameras with any perspective view are allowed to be used during the measurement process;
- Line segments are natural and plentiful in man-made architectural buildings, which makes the proposed algorithm applicable in real-world applications;
- The proposed algorithm is especially useful for *automatic* perspective distortion removal and image rectification from video sequence.

Unlike the existing image rectification techniques [24,25], we decompose the projective transformation into a chain of transformations, i.e., the pure projective transformation, the affine transformation and the similarity transformation. This process is defined as “stratification of projective rectification”. With this idea the problem can be stratified into different steps and in each step, only two or three rectification parameters have to be solved from nonlinear equations. We employ parallelism and orthogonality relationships of lines that are common and plentiful in architectural scenes, to obtain projective and then affine rectification parameters. With these parameters determined, images could be rectified up to a similarity and the relative positions, displacements and deformations of the building can be finally computed by a global scale factor.

The remainder of this paper is structured as follows: Section 2 lays the foundation for the representations and terminology that will be used in the subsequent parts of this paper and introduces the basic idea of line-based projective rectification. Section 3 provides the experimental results with both synthetic images (Section 3.1) and real videos (Section 3.2). The final conclusions are drawn in Section 4.

## 2. Methodology

Notations and mathematical operations in this study are defined as follows. Scalars are denoted as (x,a) while vectors and matrices are denoted as (x,M). ⊗ is the Kronecker product. vec(·) is used to represent the linear transformation which converts the matrix into a column vector. The cross product between vectors $a=\left(a_{1},a_{2},a_{3}\right)$ and $b=\left(b_{1},b_{2},b_{3}\right)$ can be written as matrix multiplications $a\times b={\left[a\right]}_{\times }b$, where ${\left[a\right]}_{\times }$ is defined as

(1) $${\left[a\right]}_{\times }=\left[\begin{array}{ccc} 0 & -a_{3} & a_{2}\\ a_{3} & 0 & -a_{1}\\ -a_{2} & a_{1} & 0 \end{array}\right]$$

We also introduce the basic idea of projective transformation and some important notations herein. One 3D coordinate system and two 2D coordinate systems are defined in this study: a 3D world coordinate system, where points are denoted by homogeneous coordinates with upper case letters $X={\left(X,Y,Z,1\right)}^{T}$, a 2D world planar system, where we assume the plane is on Z=0 of the world coordinate system without loss of generality, and an image plane coordinate system, where points are denoted by lower case letters $x={\left(x,y,1\right)}^{T}$. Correspondence entities from different coordinate systems have the same subscript, e.g., $L_{\lambda }\Leftrightarrow l_{\lambda }$.

A camera is modeled by the usual pinhole. The transition from the world coordinate system to the image plane coordinate system for each camera is realized through a translation followed by a rotation. Let a 3×1 translation vector $C={\left(C_{x},C_{y},C_{z}\right)}^{T}$ represent the position of the camera center in the world coordinate system and a 3×3 rotation matrix R describe the orientation of the camera by means of three consecutive rotations along the three axes Z,Y,X by respective Euler angles Γ,B,A. The parameters from C and R are called camera extrinsic parameters. As regards the intrinsic parameters of the pinhole camera, intrinsic matrix K is given by

(2) $$K=\left[\begin{array}{ccc} f_{x} & s & p_{x}\\ 0 & f_{y} & p_{y}\\ 0 & 0 & 1 \end{array}\right]$$

where $\left[p_{x},p_{y}\right]$ is the coordinate of the principle point and $\left[f_{x},f_{y}\right]$ represents the focal length of the camera in terms of pixel dimensions. *s* is the skew parameter. Once the camera parameters are determined, the camera projection matrix, defined as $P_{c}=\mathrm{KR}\left[I|C\right]=K\left[R|t\right]$, where t=RC, can be computed. The relationship between a 3D point X and its image projection x is consequently given by

(3) $$x=P_{c}X$$

Let us denote the *i*th column of the rotation matrix R by $r_{i}$. The 3D world point X on the world plane Z=0 has the form $\hat{X}=\left(X,Y,0,1\right)$ and is defined by just two coordinates *X* and *Y*. It is projected into image plane system via $P_{c}$, yielding the 2D point x

(4) $$x=K\left[\begin{array}{cccc} r_{1} & r_{2} & r_{3} & t \end{array}\right]\left[\begin{array}{c} X\\ Y\\ 0\\ 1 \end{array}\right]=K\left[\begin{array}{ccc} r_{1} & r_{2} & t \end{array}\right]\left[\begin{array}{c} X\\ Y\\ 1 \end{array}\right]=H^{\prime }\left[\begin{array}{c} X\\ Y\\ 1 \end{array}\right]$$

where the non-singular homogeneous matrix $H^{\prime }\in R^{3\times 3}$ is the projective transformation between x and $\hat{X}$. It can be seen that there is a bijection between the image plane and the world word plane. That is to say, point x on the image plane is paired with exactly one point $\hat{X}$ on the world plane by ${\left(H^{\prime }\right)}^{-1}=H$. For the purpose of clarity, we slightly abuse the notation X to denote $\hat{X}$, i.e., $X={\left(X,Y,1\right)}^{T}$. Then, the relation between X and x becomes

(5) X=Hx

Once H is obtained, any image point can be mapped into the corresponding location on the world plane Z=0. That is to say, the (X,Y) coordinate of any tracked point in the 3D world system, whether on the plane Z=0 or not, is able to be reconstructed. Thus, the only remaining problem is that of estimating the projective transformation H. Normally, H is computed directly from a set of at least four corresponding points. In this research, however, line-based geometric relationships on the world plane, such as parallelism and orthogonality, are employed to compute the projective and affine components of H.

The point $x={\left(x,y,1\right)}^{T}$ lies on the line $l={\left(a,b,c\right)}^{T}$ if and only if $x^{T}l=ax+by+c=0$. Under the point transformation X=Hx, a line transformation can be written in the form

(6) $$L=H^{-T}l.$$

On the image plane system, a vanishing point is a point where mutually parallel lines in the world system appear to converge. For different sets of lines parallel to a world plane, their respective vanishing points may lie on a same line, called a *vanishing line*, which is expressed as $l_{\infty }$ in this research. Under a projective transformation, vanishing points are finite points, and consequently $l_{\infty }$ is mapped to a finite line. Understanding the meaning of vanishing line is important because, in what follows, it is shown that the projective distortion may be removed once $l_{\infty }$ is specified.

**Figure 1:**
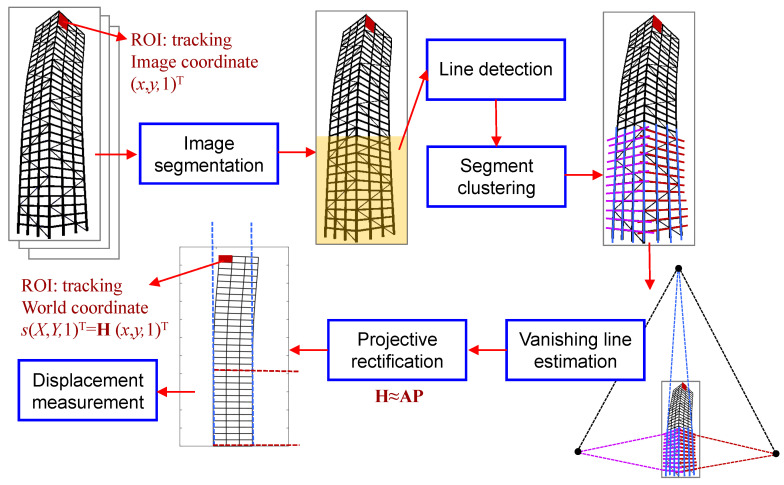
Overview of the proposed technique.

As shown in Figure 1, our proposed technique works in five steps: (1) image segmentation, (2) line detection and segment clustering, (3) vanishing line estimation, (4) stratification of projective rectification, and (5) displacement measurement. In the rest of this section, we provide technical details for steps (1)–(4). Having obtained the projective transformation H, any image-tracked point can be projected to the world plane and the displacement of this point in plane is then obtained by calculating the Euclidean distance between this point and the stationary point with a global scale factor. Since this process in step (5) is rather clear, it will not be discussed in detail in this section.

### 2.1. Image Segmentation

**Figure 2:**
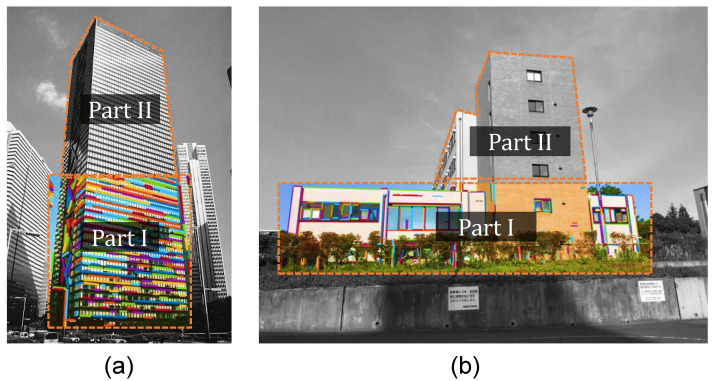
Examples of image segmentation (Line detection by [21]): (**a**) target building itself as part I; (**b**) structures from surrounding environments as part I.

Image segmentation is always an essential component in many vision-based systems. It involves partitioning images into multiple objects [17]. For the case of structural displacement measurement in this study, image segmentation is adopted first to identify the target building facade from surrounding urban areas. Next, the target building facade is divided into two parts: the (nearly) rigid region (part I) and the deformed region (part II) of the building, as shown in Figure 2.

Numerous image segmentation algorithms have been proposed in the literature for building facade segmentation. Different techniques should be chosen for different field situations: from the earliest thresholding [26], k-means clustering [27] methods to the most popular deep learning-based methods [28]. It is noteworthing that automatic building facade detection methods [29,30,31] have been well developed for geometric 3D reconstruction, and thus are more suitable for the application in the proposed vision-based measurement system.

After successful identification of the target building from the surrounding structures, line detection and projective rectification steps are restricted to part I of the segmentation. The rigid region part I is only an approximation and may vary in building types, building heights and surrounding environments. Any structures which have negligible deformations compared to the target building can be included, as shown in Figure 2b. Section 3.1 also proved that even the lower part of the target building itself (as in Figure 2a) could be set as the rigid region.

### 2.2. Line Detection and Segment Clustering

Building facades generally exhibit numerous lines that are either parallel or orthogonal to the gravity direction. Many methods have been proposed for automatic line segment detection since the 1980s, such as Canny operator [32] and Hough transform [33]. These methods are generally slow and usually combine non-contiguous line segments together, producing a lot of false detections. The recently proposed fast, parameterless line detectors, e.g., EDLines [34] and LSD [35], have shown good detection results for most types of image with enough accuracy and robustness and have been widely employed for line-based vision tasks.

Given a set of lines extracted from the building facade, the existing methods mainly rely on clustering these lines into sets of world parallel lines that converge to the same vanishing points. Commonly, the RANSAC estimation algorithm to search for concurrent lines in the image is used for segment clustering. In the RANSAC algorithm, the fitting model consists of a point and the sample is obtained by choosing randomly pairs of line segments. The intersection of each pair is computed so as to get putative vanishing points, and then the support for this vanishing point is found. The final concurrent lines result from the model with sufficient supports. This procedure was described in detail in [36]. Other segment clustering methods include identifying different groups of parallel lines in the PClines dual spaces [37] or relying on the Helmoltz principle [38].

Two groups of line segments are expected to be obtained for each of the target building facade plane by the above segment clustering strategies: the vertical lines $l_{z}$ and the horizontal lines $l_{h}$.

### 2.3. Vanishing Line Estimation

**Figure 3:**
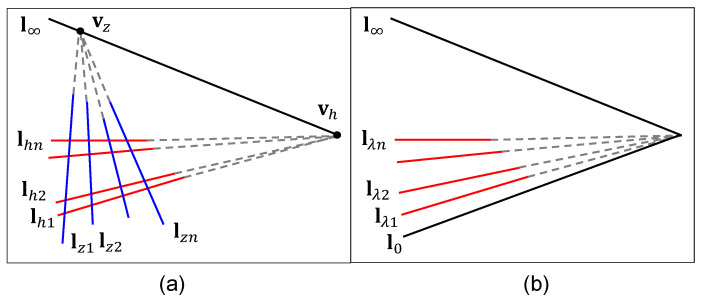
Vanishing line estimation: (**a**) method using vanishing points, (**b**) method using equally spaced parallel lines.

Normally, vanishing points at different directions $v_{h},v_{z}$, which correspond to the horizontal lines $l_{h}$ and the vertical lines $l_{z}$ respectively, can be found once the segment clustering has been determined. Two vanishing points determine a vanishing line $l_{\infty }$, i.e., $l_{\infty }=v_{h}\times v_{z}$, which is of key importance to extract geometric constraint information and remove projective distortion from the current image, as shown in Figure 3a. However, challenges in vanishing point detection arise from the inherent measurement error. The existing pixel noise, image distortion and discretization error might greatly affect the location of the vanishing point greatly, especially when camera parameters and motions are unavailable, or the vertical vanishing point is found to be infinite, when the problem becomes even harder.

If part of the horizontal lines corresponding to $l_{h}$ are equally spaced in the world coordinate system, which is easily satisfied for building facades with regular textures, an alternative vanishing line estimation method can be applied without the need of estimates of vanishing points. Consider a group of parallel lines $L_{\lambda }$ on the world plane Z=0 with the following uniform expression:

(7) $$L_{\lambda }:\;aX+bY+\lambda =0$$

This group of parallel lines may naturally be represented by the common normal vector ${\left(a,b\right)}^{T}$ and different lines have different values of λ. With this preparation and further considering Equation (6), one could obtain the corresponding lines in the image plane as

(8) $$l_{\lambda }=H^{T}L_{\lambda }=H^{T}\left(\begin{array}{cc} 0 & a\\ 0 & b\\ 1 & 0 \end{array}\right)\left(\begin{array}{c} \lambda \\ 1 \end{array}\right)=V\left(\begin{array}{c} \lambda \\ 1 \end{array}\right)$$

where V is a non-singular 3×2 matrix and determined to scale. It has been proved that the first column of V stands for the vanishing line $l_{\infty }$ and the second column of V represents the line $l_{0}$[36], as shown in Figure 3b. Consequently, once the matrix V is determined, the vanishing line can be obtained. The process of estimating the matrix V through line correspondences $\left(\lambda_{i}\Leftrightarrow l_{\lambda i},i=1,\cdots ,n\right)$ is similar to that of estimating homography H through point correspondences: we begin with the basic Direct Linear Transformation (DLT) algorithm to compute the initial value of V and then turn to the iterative minimization method to find the optimal estimate of V.

**DLT algorithm.** Now, swapping both sides of Equation (8) and multiplying them by ${\left[l_{\lambda }\right]}_{\times }$ yields

(9) $${\left[l_{\lambda }\right]}_{\times }V\left(\begin{array}{c} \lambda \\ 1 \end{array}\right)=0$$

Using the Lemma in [39], one has

(10) $$\left(\left(\lambda ,1\right)⊗{\left[l_{\lambda }\right]}_{\times }\right)\cdot \mathrm{vec}\left(V\right)=0$$

If we set $M_{i}={\left(\lambda_{i},1\right)}_{i}⊗{\left[l_{\lambda i}\right]}_{\times }$ and a=vec(V), Equation (10) can be re-written as

(11) $$M_{i}a=0$$

Each line correspondence gives rise to two independent equations of a. Given a set of three such line correspondences, we obtain a set of equations Ma=0, where M is the matrix of equation coefficients built from the matrix rows $M_{i}$. Note that the M has rank 5, and thus has a 1-dimensional null-space which provides a solution for a. Generally, a is obtained by the singular value decomposition (SVD) of M, i.e., the unit singular vector corresponding to the smallest singular value of M is the solution a.

**Iterative minimization.** With the initial value of V obtained from the DLT algorithm, the next step is to get the best estimate of V by minimizing an appropriate geometric cost function with the use of iterative techniques. Herein, the cost function is selected as

(12) $$\sum_{i=1}^{n}\sum_{j=1}^{m}d{\left(x_{ij},l_{\lambda i}\left(V\right)\right)}^{2}$$

where d(x,l) represents the perpendicular distance from a point x to the line l. $l_{\lambda i}\left(V\right)=V{\left(\lambda_{i},1\right)}^{T}$ and $x_{ij}$ is the *j*th (j=1,⋯,m) point on the *i*th line on the image plane. In this research, the cost function in Equation (12) is minimized using the Levenberg–Marquardt algorithm.

Grouping the equally spaced lines $l_{\lambda }$ from $l_{h}$ can also be addressed by the RANSAC algorithm. For a complete exposition of this procedure, the reader is referred to [36].

### 2.4. Stratification of Projective Rectification

**Figure 4:**
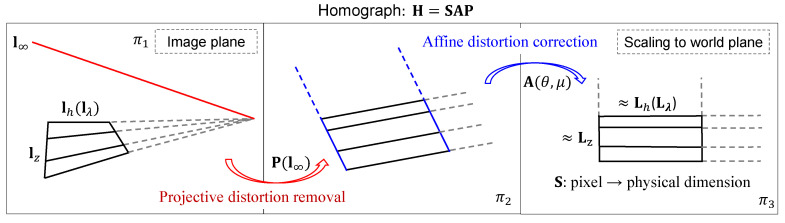
Image rectification: from projective distortion removal to affine distortion correction.

The goal of projective rectification is to remove the projective/affine distortion in the original image plane ($\pi_{1}$) to the extent that similarity properties (angles, ratios of lengths) could be measured on the transformed plane ($\pi_{3}$). In most of the previous research, this was directly completed by specifying the position of at least four reference points. In this research, however, metric structure recovery is stratified so that the pure projective distortion is removed first and then the affine distortion is corrected, by using line-based constraints (i.e., parallelism and orthogonality). The idea of stratified projective rectification is illustrated in Figure 4.

For the group of invertible n×n matrices with real elements, the general linear group on *n* dimensions can be expressed as GL(n). The homography is a quotient group of GL(3), giving PL(3) (e.g., det(H)=1). According to [40], subgroups of PL(3) include the affine group and the similarity group. As a result, the projective transformation matrix H could be uniquely decomposed into three matrices [41]

(13) H=SAP

where P is a ‘pure projective’ transformation, A is an affine transformation and S is a similarity transformation, respectively.

The ‘pure projective’ transformation P can be obtained directly by the vanishing line $l_{\infty }={\left(l_{1},l_{2},l_{3}\right)}^{T}$ of the image plane as

(14) $$P=\left(\begin{array}{ccc} 1 & 0 & 0\\ 0 & 1 & 0\\ l_{1} & l_{2} & l_{3} \end{array}\right)$$

Under a projective transformation, $l_{\infty }$ is mapped to a finite line while under an affine transformation, $l_{\infty }$ is not mapped to a finite line but remains at infinity. In other words, if the imaged line at infinity is the line $l_{\infty }={\left(l_{1},l_{2},l_{3}\right)}^{T}$, then provided $l_{3}\neq 0$, the above `pure projective’ P maps the line $l_{\infty }$ on $\pi_{1}$ back to the line ${\left(0,0,1\right)}^{T}$ on a new plane $\pi_{2}$, where no projective distortion exists. This is directly evident from Equation (6) that $P^{-T}{\left(l_{1},l_{2},l_{3}\right)}^{T}={\left(0,0,1\right)}^{T}$.

The affine transformation in Equation (13) is represented by an upper-triangular matrix

(15) $$A=\left(\begin{array}{ccc} \frac{1}{\beta } & -\frac{\alpha }{\beta } & 0\\ 0 & 1 & 0\\ 0 & 0 & 1 \end{array}\right)$$

where parameters α and β represent the circular points of the plane, which are invariant under similarity transformations. The circular point is a pair of complex conjugate points on the vanishing line. Circular points are transformed from similarity coordinates ${\left(1,\pm i,0\right)}^{T}$ to affine coordinates ${\left(\alpha \mp \beta i,1,0\right)}^{T}$ by the affine transformation A.

Finally, the similarity transformation S is expressed by

(16) $$S=\left(\begin{array}{cc} sR & t\\ 0^{T} & 1 \end{array}\right)$$

where R is a 2×2 orthogonal rotation matrix, *s* an scaling factor, t a translation 2-vector and 0 a null 2-vector.

We ignore the metric part S of H in the solving process of projective rectification and only recover the non-metric part N=AP. Under a stratified rectification scheme, the two components, P and A of N, are recovered step by step, as follows.

#### 2.4.1. Projective Distortion Removal

Based on Equation (14), the pure projective transformation P is only determined by the vanishing line $l_{\infty }$. As mentioned in Section 2.3, two methods may be used to determine $l_{\infty }$: the method using vanishing points $v_{h},v_{z}$ and the method using equally spaced parallel lines $l_{\lambda }$. We summarize details for each of the methods to obtain the transformation P in Table 1 (Part 1).

#### 2.4.2. Affine Distortion Correction

Affine distortion correction makes angles in the rectified image equal to angles in the world. In this research, the affine distortion correction is accomplished in two steps, according to the following decomposition of the affine transformation A

(17) $$A\approx A_{2}A_{1}=\left(\begin{array}{ccc} \mu & 0 & 0\\ 0 & 1 & 0\\ 0 & 0 & 1 \end{array}\right)\left(\begin{array}{ccc} 1 & -\cot\left(\theta \right) & 0\\ 0 & 1 & 0\\ 0 & 0 & 1 \end{array}\right)$$

where θ is the angle between the directions of $l_{z}^{\prime }$ and $l_{h}^{\prime }$, or say $v_{h}^{\prime }$ and $v_{z}^{\prime }$, and μ is viewed as the aspect ratio, which corresponds to the relative scale of the horizontal and vertical directions. In fact, the line-based information we have used to date only provides the constraint for θ in $A_{1}$, while leaving the ambiguity caused by the unknown aspect ratio μ in $A_{2}$. When we are most concerned with rectification of building facades—where rectangular structures always exist, such as the facade outline or windows—the aspect ratio μ can be easily acquired by the known width to height ratio of those rectangular structures. μ is also to be resolved from partial knowledge of the internal camera parameters in [42]. The above procedure of affine distortion correction is also described in detail in Table 1 (Part 2).

## 3. Experimental Case Studies

To experimentally validate the proposed method of vision-based displacement measurement, two case studies are presented in this section. In the first synthetic experimental test, the projective rectification was conducted directly based on the lines extracted from the regions of the target building itself. The appropriate regions to extract line segments for image rectification are discussed and verified for a typical high-rise building. In the second real-recorded case, line information was directly read from a reference board. We showed that while the scenes suffered, a strongly changing field of view taken by a hand-held camera, the proposed method still demonstrates a sub-pixel measurement accuracy.

### 3.1. Synthetic Experiments

#### 3.1.1. A 30-Story Building Model

**Figure 5:**
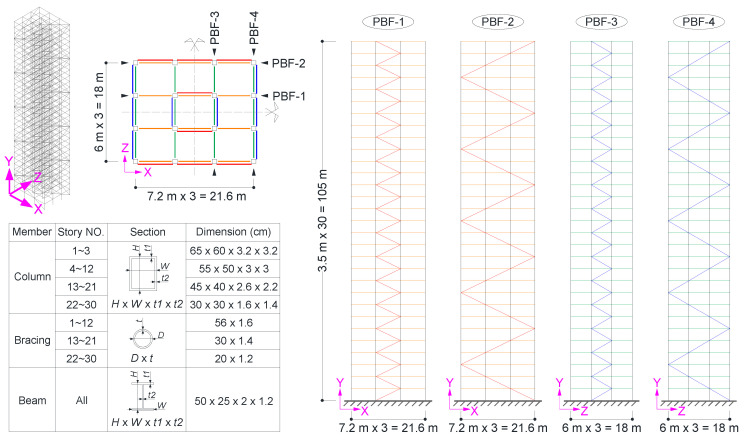
Basic information of the 30-story building model.

First, we evaluate the feasibility, accuracy and robustness of the proposed method using synthetic images, since no real recorded video of building structures subjected to earthquake ground motion was available at the present time. The Synthetic images were generated by four synthetic cameras observing a 3D finite element model of a 30-story building structure. The structure is a braced frame, with a height of 105 m, as shown in Figure 5. The layout and the member section of the structure are detailed in the figure. The load on the structure was modeled as seismic masses lumped at the floor levels. The finite element model of the structure was established in the ANSYS program [43], whereas the beam, column, and brace were simulated by the beam188 element, while the lumped mass was simulated by the mass21 element. Within each floor, the translational degrees of freedom of the nodes were coupled to comply the rigid floor assumption. The basic dynamic properties of the structure, including the natural period and the shape of the first six natural modes, are listed in Table 2. Specifically, the fundamental period $T_{1}$ = 2.47 s is in compliance with the engineering practice [44,45,46].

**Figure 6:**
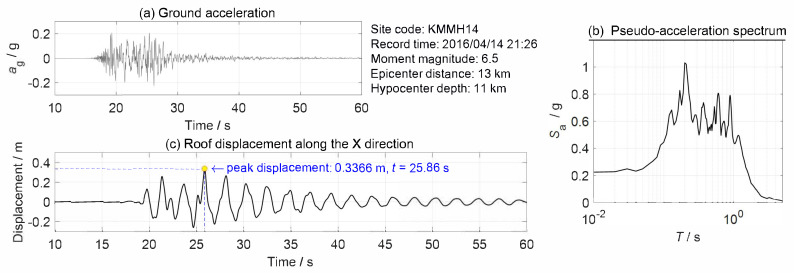
Earthquake excitation and displacement response of the building: (**a**) time history of the ground acceleration, (**b**) pseudo-acceleration spectrum, and (**c**) roof displacement.

The structure was subjected to a ground acceleration along the X direction (see the world coordinate in Figure 5). The seismological information and the pseudo-acceleration spectrum of the excitation is shown in Figure 6a,b, respectively. The lateral displacement history of the roof is shown in Figure 6c. From Figure 6c, the peak lateral displacement of the roof is 0.3366m. The accordant story drift angle (ratio of the peak roof displacement to the structural height) is about 1/312 rad. Since there is a great deal of concern in the peak displacement response of the building during an earthquake event, only the corresponding structural displacements at t = 25.86 s were extracted and used to generate the synthetic images in this example.

#### 3.1.2. Image Generation

**Figure 7:**
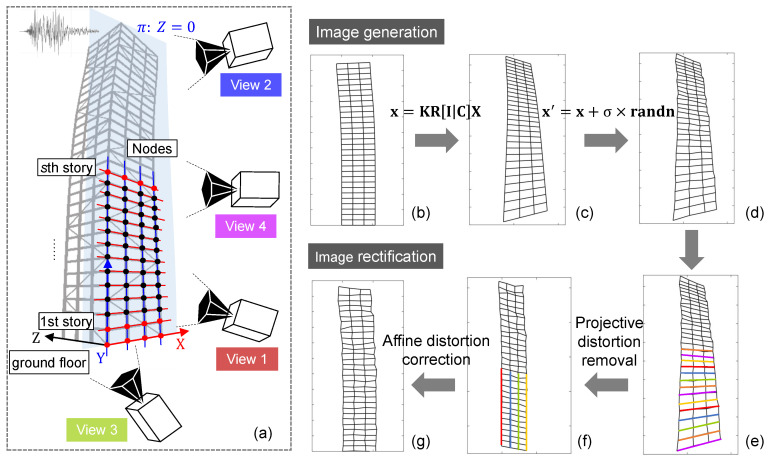
Processes of image generation and image rectification (building deformations are drawn to a scale of ten for enhanced visualizations): (**a**) positions of the camera, (**b**) world plane Z = 0, (**c**) synthetic image after camera projection, (**d**) image perturbed with noise, (**e**) image with line detection, (**f**) image after projective distortion removal, (**g**) image after affine distortion correction.

**Figure 8:**
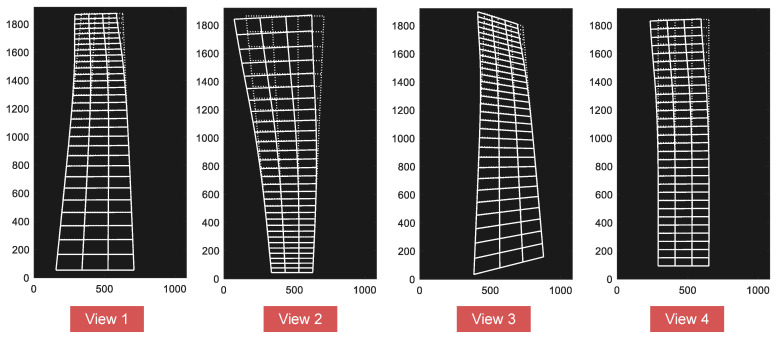
Synthetic views used in this example (building deformations are drawn to a scale of ten for enhanced visualizations).

Four artificially created views were generated from four separate synthetic cameras, as shown in Figure 7a and Figure 8. For each view, a virtual pinhole camera with the image size of 1920×1080 pixels was modeled, with different camera positions and rotations. The camera was then adjusted by different focal lengths so that all line segments were in its field of view.

The image coordinate system is assumed to be Euclidean coordinates with equal scales in both axial directions ($f_{x}=f_{y}=f$) and *s* is set to zero in this example. Details of the camera parameters for each synthetic view are listed in Table 3. It is noteworthy that the above camera parameters were only used for synthetic image generation. Displacement measurement using the proposed method requires no knowledge of camera parameters.

Line segments were generated by placing the nodes of beam elements at world plane π:Z=0 as endpoints, see Figure 7a. The line segment l passing through two image endpoints $x_{1}$ and $x_{2}$ is then obtained by $l=x_{1}\times x_{2}$, where × is the vector product. If more than two points are provided, the method of orthogonal regression is used to estimate the line that gives the best fit to those points. The linear projection of 3D world line segments onto the image plane can also be achieved directly using Plücker coordinates. This procedure was described in detail in [47]. Note that only the nodes close to the ground floor with trivial displacements are limited for use in line generation and projective rectification. The vanishing line was estimated based on the equally spaced parallel lines generated by the nodes from ground floor to the *s*th story, s=8−22.

To investigate the robustness of the proposed method, the coordinates of the endpoints in the image plane were additionally perturbed with independent and identically distributed Gaussian noise (Figure 7d), with a standard deviation of σ=0.5 pixel and σ=1.0 pixel, respectively. The relationship between the pixel coordinate and the physical dimension in the world system is bout 0.040 m/pixel-0.074 m/pixel (bottom-top) in View 1. Monte Carlo simulations with 1000 trials for each noise level were performed under the above setup.

#### 3.1.3. Measurement Results

We introduce the root mean square error (RMSE) between the image-estimated nodal coordinates in X-direction ($X_{i},i=1,\cdots ,N$) of the structure after image rectification and the corresponding ground truth coordinates ($X_{Gi}$) obtained by ANSYS in each image as follows

(18) $$RMSE=\sqrt{\frac{1}{N}\sum_{i=1}^{N}{\left(X_{i}-X_{Gi}\right)}^{2}}$$

The proposed method was evaluated and compared with the widely used, point-based image rectification technology, where the projective transformation H in Equation (5) is directly estimated by minimizing a robust maximum likelihood cost function using matched points [40]. Two cases, in which enough points (both black and red nodes in Figure 7a) and sparse points (only the red nodes in Figure 7a) were employed for projective rectification, were taken into consideration herein.

**Figure 9:**
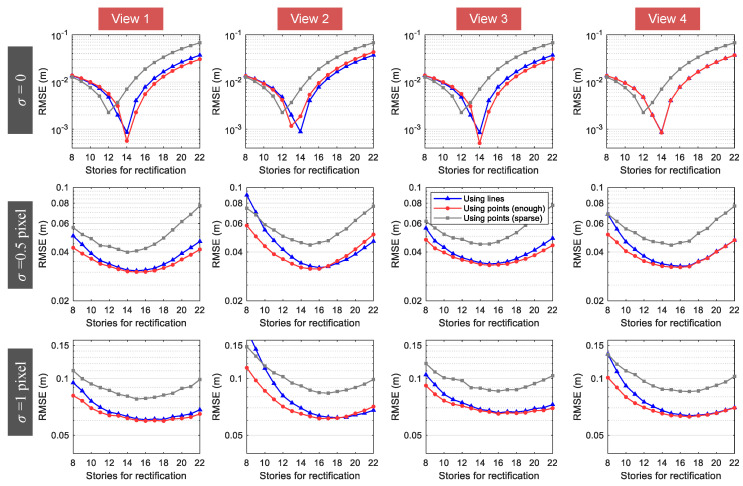
Measurement results for each view with different noise levels.

**Figure 10:**
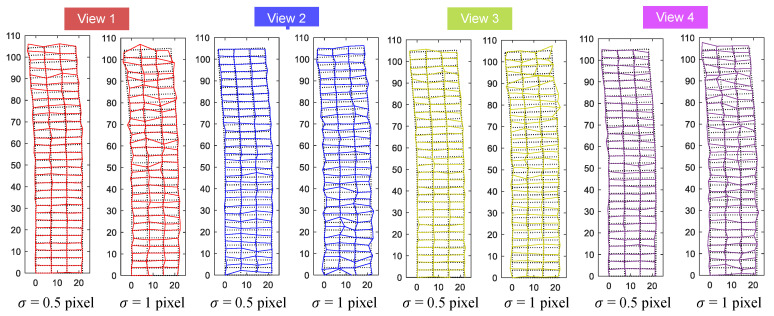
Rectified images from each view with different noise levels (Building deformations are drawn to a scale of ten only for enhanced visualizations).

Results of the synthetic experiment are summarized in Figure 9 and typical line-based rectified images from each view with different noise levels are referred to Figure 10.

The RMSEs in Figure 9 were computed based on the overall nodes on each view. The results in the figures show that, when an appropriate region from the target building was used for projective rectification, the RMSE of measured displacements was below $10^{-3}$ m if no pixel noise exists in the image. For the cases with noises, the lowest RMSE was about 0.03 m for σ=0.5 pixel, and 0.06 m for σ=1 pixel, respectively. It is easily observed that there is an optimal value for the number of stories that can be used for projective rectification. The reason for this is that, on one hand, the more information (lines/points) is used for image rectification, the greater the accuracy that can be achieved. On the other hand, the displacements of the lines/points positioned at higher stories of the target building were nontrivial under ground motion, and thus inappropriate to be involved in the projective rectification step. From the noise-free case, the measurement error with RMSE = 37 mm for s=22 was shown to be 46 times larger than that with RMSE = 0.8 mm for s=14. It appeared that the accuracy difference became less significant as the levels of noise went up. According to Figure 9, values of *s*, which led to the measurement errors that did not deviate much from the minimum, can range from 10 to 20 stories for both σ=0.5 pixel and σ=1 pixel. It is thus proved that the abundant line information in the region from the ground to about 1/3–2/3 height of the building itself might also be able to be used for projective rectification in this case. However, when the targeting building itself was used, the measurement results became very sensitive to the image noise. Measurement errors were 0.2% (0.0008 m/0.337 m) for no noise, 9% (0.03 m/0.337 m) for σ=0.5 pixel, and 18% (0.06 m/0.337 m) for σ=1 pixel. As we can see, when the pixel noise approaches one, the measurement results seem to be unacceptable. In such cases, a camera with higher resolution should be used, or a more strictly rigid region around the building should be applied for projective rectification.

Figure 9 also reports that our line-based method and the normally used, point-based method with the same number of points applied for image rectification behaved nearly identically, with the latter being slightly more accurate for small value of *s*. This was attributed to the accuracy degradation of the estimated orthogonal lines $l_{v}$, which became more sensitive to the noise, with fewer points used for orthogonal regressions. Inaccurate $l_{v}$ disturbed the precision of the affine distortion correction and resulted in errors to the final measurements in X-direction. A possible solution to circumvent this issue is to alternatively identify the normal n of the ground plane by plane detection techniques [48,49]. Since n is parallel to $l_{v}$, it can be directly used for affine distortion correction in Section 2.4.2. This process will be discussed in detail in further research. Moreover, it should be pointed out that for low-texture scenes with man-made building facades in urban environments, line features are often more abundant and reliable than point features. If only sparse points were provided, measurement errors would significantly increase, as depicted in Figure 9 for the point-based rectification method, where the RMSE results change from the red lines to the gray lines. In such cases, using line features for displacement measurement is much more feasible and practicable.

**Figure 11:**
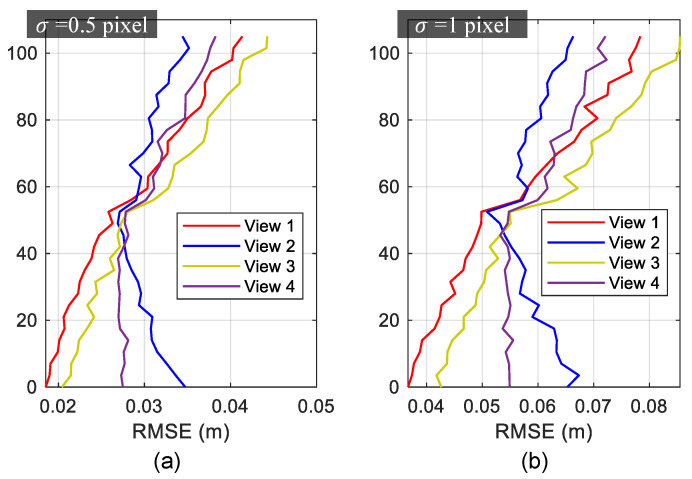
RMSE for each story: (**a**) σ=0.5 pixel (**b**) σ=1 pixel.

For all of the four different views, the results of view 2 were the least accurate ones based on the overall RMSE depicted in Figure 9. This might be due to the fact that the corresponding camera was farthest from the region used for projective rectification among all of the synthetic views and this leads to a likely overall inaccuracy. If the RMSE for each story was calculated separately, view 2, on the other hand, resulted in the highest measurement accuracy for the upper region of the building, as shown in Figure 11. Figure 11 also shows that the measurement accuracy for the upper region decreased as the camera’s position was down in the Y direction (refer to $C_{y}$ in Table 3). That is to say, different regions of the target building had varying measurement accuracy for the image with perspective view, and the structural region nearest to the camera’s position attained the greatest precision.

Attention should also be paid to view 4, since this view went through the least projective distortion but did not yield sufficiently accurate overall measurement results, as expected. The reason behind this is that focal length of this view had to be changed to 500 pixels so that all lines/points were in its field of view, while it was able to be set to 600 pixels for the other views. Based on the above observations, cameras are suggested to be positioned close to the ground/rigid regions of the structure if the accuracy of the overall motion is required, or near to the top area of the building if the roof displacement is more concerned, with powerful zoom lens and a large enough field of view for accurate measurement.

### 3.2. Experiments on Real Video Sequences

#### 3.2.1. Experiment Test Setup

Since a high-rise tested structure was difficult to obtain due to the limitations of experimental facilities, in this experiment, we detected lines for projective rectification directly from a reference board instead of the target structure itself.

**Figure 12:**
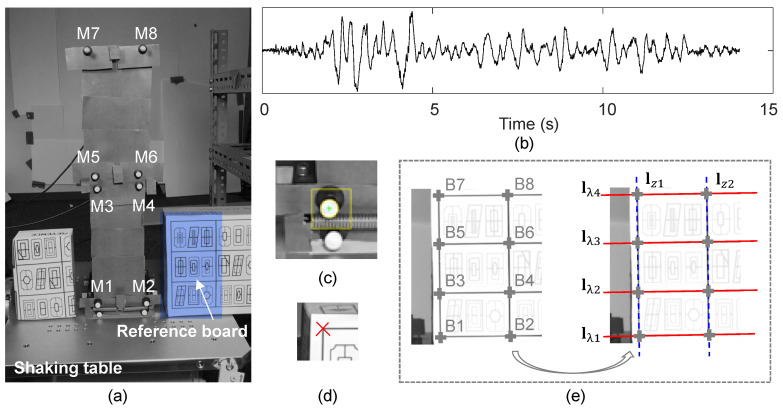
Experiment test setup: (**a**) overview of the two-story base-isolated structure with artificial tracking markers, (**b**) scaled earthquake ground motion acceleration record, (**c**) circular-based tracker, (**d**) correlation-based tracker, (**e**) reference board and lines extracted for projective rectification.

Figure 12a shows the overview of the experiment. The two-story base-isolated structure is with 140 mm width and 400 mm height. Rge asses of each story are about $m_{0}=2.100$ kg (base story), $m_{1}=3.247$ kg (first story) and $m_{2}=1.531$ kg (second story). The stiffness is about $k_{1}=2.3\times 10^{4}$ N/m and $k_{2}=7.5\times 10^{3}$ N/m for the first story and second story, respectively. In the base story, a BSG-H10 slider with frictional interfaces and tension springs with stiffness of $k_{s}=2.3\times 10^{3}$ N/m are assembled as a seismic isolator for this structure. The structure is bolted on the shaking table with the size of 400 × 400 mm. The scaled earthquake record of 2016 Kumamoto earthquake in Figure 12b is selected as the ground motion. The data of this record are from K-NET and KiK-net of Japanese NIED strong-motion seismograph networks [50].

To validate the *relative displacement* results measured by the vision-based system, four laser transducers were installed on a shelf close to the shaking table as reference sensors. Absolute displacements and accelerations of the shaking table, the base story, the first story and the second story of the structure were additionally recorded by the reference laser transducers and accelerometers, respectively. Let $a_{g},a_{0},a_{1},a_{2}$ denote the absolute accelerations of the shaking table, the base story, the first story and the second story of the structure, and $u_{g}^{*},u_{0}^{*},u_{1}^{*},u_{2}^{*}$ denote the absolute displacement. It should be noted that the relative displacements $u_{0},u_{1},u_{2}$ with respect to the ground motion for each story were directly measured by the vision-based system, while they were reconstructed by the absolute displacements $u_{g}^{*},u_{0}^{*},u_{1}^{*},u_{2}^{*}$ from the laser transducers via $u_{0}=u_{0}^{*}-u_{g}^{*},u_{1}=u_{1}^{*}-u_{g}^{*},u_{2}=u_{2}^{*}-u_{g}^{*}$.

A hand-held iPhone 7 equipped with 28-mm lenses (12 MP, f/1.8,1/3″) was used for displacement measurement of the structure during the input of the seismic ground motion. Artificial white circular markers (M1-M8) glued to the base, first and second floor of the structure were tracked throughout the video by computing the coordinates of their gravity centers on the image plane (see Figure 12a,c).

Four equally spaced horizontal lines ($l_{\lambda 1}∼l_{\lambda 4}$) and two vertical lines ($l_{z1},l_{z2}$) were extracted by tracking the group of points (B1–B8) provided on the reference board, as shown in Figure 12e. B1–B8 were tracked based on the correlation-based template matching method. The method using equally spaced parallel lines to estimate the vanishing line was applied, since available vertical lines were limited.

**Figure 13:**
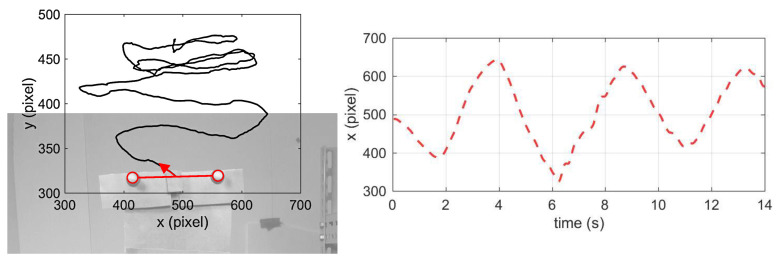
Trajectory of the markers in the second story before image rectification.

The iPhone was subjected to large motions, such as translations and rotations, while the structure was subjected to the seismic ground motion. Tracking results of the markers without any image rectification are shown in Figure 13, taking the trajectory of the second story (the mean value of M7-M8’s tracking coordinates (in pixel)) as an example.

As is seen, real displacements of the structure were overwhelmed by the strongly changing field of view caused by the moving camera and seemed hard to distinguish from their overall trajectories if no image rectification was involved.

#### 3.2.2. Measurement Results

**Figure 14:**
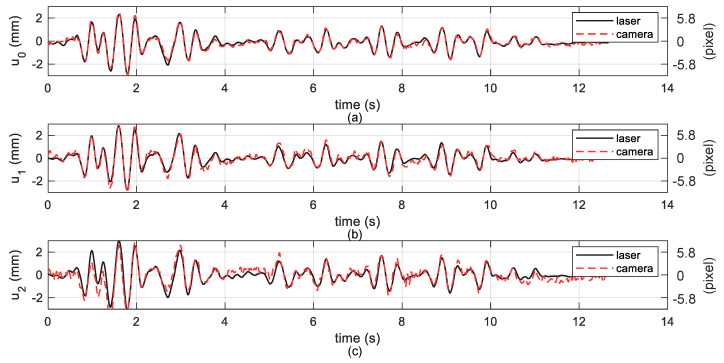
Measured results from the proposed method: (**a**) the base story displacement $u_{0}$, (**b**) the first story displacement $u_{1}$, (**c**) the second story displacement $u_{2}$.

The final vision-based displacement measurement results using the proposed line-based projective rectification method are graphically shown in Figure 14.

**Figure 15:**
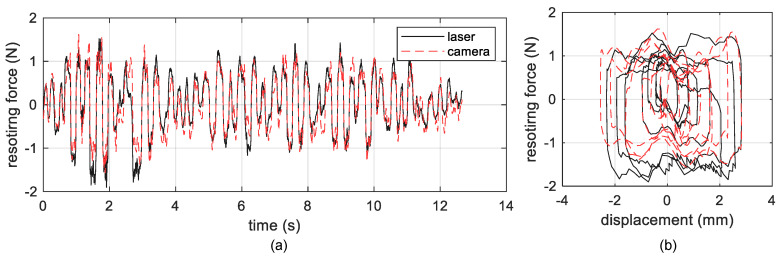
Frictional force *F* computed by the measured displacement and acceleration data: (**a**) time history of *F*, (**b**) $u_{0}$ vs. *F*.

It is clear that the proposed method provides the measurement results with comparable accuracy to that obtained by referenced laser transducers. Next, the accuracy of the displacement measurement was additionally assessed by computing the nonlinear frictional force on the base interfaces of the structure from the fusion of measured displacement data and acceleration data using the following equation

(19) $$F=-\left(a_{1}m_{1}+a_{2}m_{2}+a_{0}m_{o}\right)-k_{s}u_{0}$$

where $a_{1},a_{2}$ and $a_{0}$ are the measured accelerations of the first, second and base story, respectively. Figure 15 demonstrates that the vision-based measurement results agree well with the laser-based results in system force estimation. With the aid of successfully measured displacement data, more key physical performances of the mechanical system can be investigated and the perdition of system’s further behavior for prognosis in structural health monitoring is thus able to be delivered.

**Figure 16:**
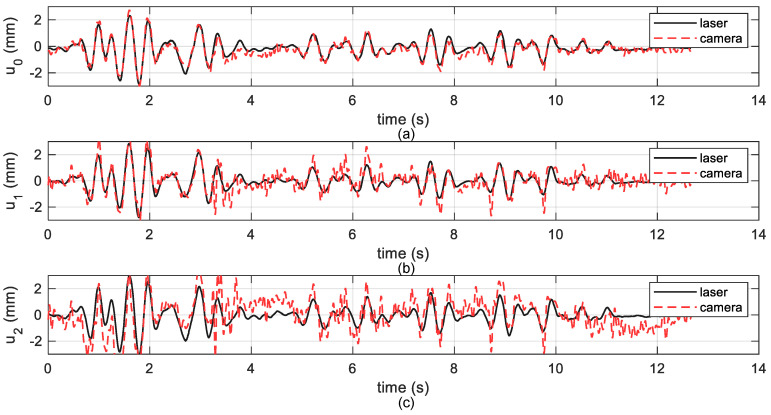
Measured results based on point-based projective rectification method: (**a**) the base story displacement $u_{0}$, (**b**) the first story displacement $u_{1}$, (**c**) the second story displacement $u_{2}$.

To further validate the effectiveness of the proposed method, the displacement measurement results obtained by the point-based projective rectification method, in which the same points B1-B8 were specified to be used to compute the maximum likelihood estimation of H in Equation (5), are presented in Figure 16.

Comparing the results from different methods for projective rectification, the line-based method has a much better performance in terms of algorithm accuracy. In theory, the line-based rectification and point-based rectification should be equivalent, as they were provided with the same information. Nevertheless, it is found that the line-fitting is generally more noise-resistant than merely point detection, especially for the cases when feature information is limited. For a complete exposition of this inference and special attentions which should be paid when using the line-based method, the reader is referred to [51].

Finally, we computed the RMSE between the vision-based displacement measurements and the laser references. The RMSE is plotted for each video frame in pixel.

**Figure 17:**
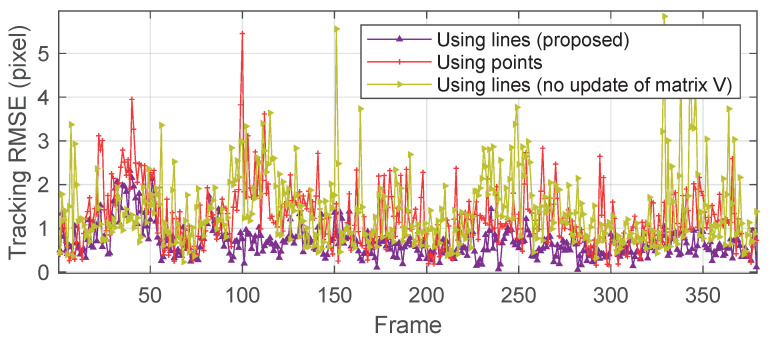
Tracking RMSE for each frame.

According to Figure 17, it is seen that errors in most frames of our line-based method are below one pixel. The average RMSE is 0.68 pixel per frame while 1.25 pixel for the point-based method. The measure error is about 8% (0.68 pixel/8.08 pixel) for the proposed approach, which is satisfied and acceptable for dynamic displacement measure. Comparing the tracking history of RMSE also allows us to quantitatively assess the contribution of the matrix V update strategy by the iterative minimization techniques proposed in Section 2.3. When we re-ran the algorithm without the update of V using only the value estimated by the DLT algorithm, the resulting average error increased to 1.41 pixel per frame. These results demonstrate that the accurate estimation of the matrix V also provides an apparent boost to measuring performance.

## 4. Conclusions and Discussion

A new vision-based system for seismic displacement measurement of building structures is developed in this research. The technique only requires line-based geometric relationships on the building facade plane to be extracted from a single, uncalibrated, perspective view. To remove the projective/affine distortion in the original image, stratification of the projective rectification technique, which is accomplished by employing parallelism and orthogonality of lines to compute the projective and affine components of the transformation sequentially, is introduced in this research. Synthetic examples and experiments on real video sequences are explored. The accuracy of displacement estimates and the robustness to image noise of the proposed method were validated.

To date, we have shown some advantages of the proposed method in projective rectification and structural displacement measurement. In practice, however, several attentions should be paid and some challenges still remain open:

- Only step (3)–(5) in Section 2 were validated via experimental case studies in this study, since no real recorded video for seismic-induced motion measurement of building structures was available at the present time. A vision-based system with the newly released Canon EOS R5 camera (4 K at 120 fps) has already been incorporated into a structural health monitoring system of a high-rise building and the proposed method is expected to contribute to the further research;
- Although sub-pixel level accuracy was attained in this study, the real application of this image-processing technique might be inferior to the laboratory precision since, in the real world, there exists not only pixel noise, but also image distortion as well as line segment extraction error, which make the problem much more challenging;
- When using vanishing points to rectify the image, any horizontal/vertical parallel lines, coplanar or not, can be involved in the algorithm, while the method using equal spaced lines requires coplanar parallel lines;
- When structures are subjected to out-of-plane motion, the proposed method is still applicable. To measure in three dimensions, image rectifications with respect to two mutually orthogonal planes of the building, e.g., Z=0 and X=0 in Figure 7a, should be employed from a single image. Therefore, three dimensional measurements may incur the trade-off problem between measurement resolution and the field of view. A higher resolution, such as 4 K (e.g., 3840×2160), is suggested to be set for accurate measurement.

## Figures and Tables

**Table 1:** Stratification of projective rectification.

**Input:** Video frame *i* is available to read	
Image segmentation to identify the target building facade F ← Section 2.1	
Line detection and segment clustering: $l_{v},l_{h}$ in Part I of F ← Section 2.2	
Method using equally spaced parallel lines:	Method using vanishing points:
**Part 1**: Projective distortion removal	Projective distortion removal
1. Group $l_{\lambda }$ from $l_{h}$ ← Section 2.3	1. Obtain vanishing points $v_{h},v_{z}$ from $l_{h},l_{z}$ ← Section 2.2
2. $l_{\infty }\leftarrow V\leftarrow l_{\lambda }$ in Section 2.3	2. $l_{\infty }=v_{h}\times v_{z}$
3. $P\leftarrow l_{\infty }$ in Section 2.4.1	3. $P\leftarrow l_{\infty }$ in Section 2.4.1
4. $x^{\prime }\leftarrow Px$ in I, $l_{z}^{\prime }\leftarrow P^{-T}l_{z}$, $l_{v}^{\prime }\leftarrow P^{-T}l_{v}$	4. $x^{\prime }\leftarrow Px$ in I, $v_{h}^{\prime }\leftarrow Pv_{h}$, $v_{z}^{\prime }\leftarrow Pv_{z}$
**Part 2**: Affine distortion correction	Affine distortion correction
1. $A_{1}\leftarrow \theta \leftarrow l_{z}^{\prime },l_{v}^{\prime }$ in Section 2.4.2	1. $A_{1}\leftarrow \theta \leftarrow v_{h}^{\prime },v_{z}^{\prime }$ in Section 2.4.2
2. $A_{2}\leftarrow \mu \leftarrow$ Rectangular structures	2. $A_{2}\leftarrow \mu \leftarrow$ Rectangular structures
3. $X\approx A_{2}A_{1}x^{\prime }$ (with global scale factor)	3. $X\approx A_{2}A_{1}x^{\prime }$ (with global scale factor)
**Output:** Displacements d=ΔX, then go to the next video frame i+1	

**Table 2:** Dynamic property of the building.

Order	Type	Period/s	Mode 1	Mode 3	Mode 5	Mode 2	Mode 4	Mode 6
1	Translational-Y	2.470	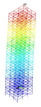	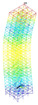	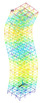	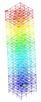	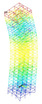	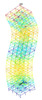
2	Translational-X	2.251						
3	Translational-Y	0.736						
4	Translational-X	0.709						
5	Translational-Y	0.392						
6	Translational-X	0.387						

**Table 3:** Camera parameters of each view.

View	Extrinsic Parameters						Intrinsic Parameters		
	Camera Position			Camera Rotation			Focal Length	Principal Point Coordinate	
	(m)			(rad)			(pixel)	(pixel)	
	$C_{x}$	$C_{y}$	$C_{z}$	A	B	Γ	f	$p_{x}$	$p_{y}$
1	15	30	–30	–0.2	0	0	600	539.5	959.5
2	15	75	–30	0.2	0	0	600	539.5	959.5
3	0	30	–30	–0.2	0.2	0	600	539.5	959.5
4	15	52	–30	0	0	0	500	539.5	959.5
